# Developing developmental cognitive neuroscience: From agenda setting to hypothesis testing

**DOI:** 10.1016/j.dcn.2015.12.011

**Published:** 2015-12-23

**Authors:** Wouter van den Bos, Ben Eppinger

**Affiliations:** a Center for Adaptive Rationality, Max Planck Institute for Human Development, Berlin, Germany; b Department of Psychology, TU Dresden, Dresden, Germany

## 1 Heuristic models, agendas and analogies

“There is nothing more practical than a good theory”

(Lewin, 1951)

In this issue of Developmental cognitive neuroscience Shulman and colleagues (n.d.) and Nelson and colleagues (n.d.) present two heuristic models of cognitive development. Shulman and colleagues review the current evidence in favor of dual systems (DS) models, which suggests that enhanced risk taking in adolescents is the consequence of an imbalance between an early maturing motivational system involved in reward processing and a later maturing cognitive control system. They conclude with the viewpoint that the current literature seems to reaffirm the usefulness of these models. In a similar fashion, Nelson and colleagues (n.d.) presented an updated version of the social information processing model (SIP), a heuristic framework which links facets of social development (ranging form infant caregiver interactions to intimate relationships during adolescence) with functional changes in the developing brain.

Models are one of the central instruments of modern science (e.g. the double helix model of DNA, the billiard ball model of a gas, or the mind as a computer). However, not all models are alike and different types of models serve different functions in the process of scientific discovery (Frigg and Hartmann, 2006). The current models of adolescent brain development, including the ones presented in this issue, are often labeled as *heuristic models* (Casey, 2014, Crone and Dahl, 2012, Nelson et al., n.d., Richards et al., 2013, Shulman et al., n.d.). However, it is not always clear what heuristic models are and what role they have. It is currently unclear if, and how, different heuristic models can be meaningfully compared, or to what extend they aid the formulation of testable hypotheses. Moreover, although there are many results that can be interpreted as being consistent with heuristic models, we will argue that such comparisons have only limited value and may even hamper further investigation of the underlying developmental mechanisms.

In this comment we provide a critical review of heuristic models, making specific references to the DS and SIP models, focusing on their ability to move the field of developmental cognitive neuroscience forward toward a mechanistic understanding of neural development. We aim to make a contribution to the debate around models by (1) providing further conceptual clarification and a framework to evaluate different types of models, and (2) by highlighting the benefits of a stronger commitment to cognitive models in order to generate testable hypotheses and integrate different levels of analyses (including neuroscience). First, we discuss the role of *heuristic models* in science as frameworks for inspiration and research agenda setting. Although heuristic models are by nature simplistic, we will suggest several principles that can be used to evaluate them. Next we discuss one direction that could be taken to foster the transition from *heuristic models* to *cognitive neuroscience models*, from agenda setting to hypothesis testing.

### 1.1 Heuristics and the context of discovery

The classic distinction between “context of discovery” and “context of justification” (Popper, 1959, Reichenbach, 1938) provides a good starting point in organizing the debate on the different models of adolescent brain development. That is the distinction between the context in which new ideas or hypotheses are generated, and the context in which those are defended. For instance, reading a novel (e.g. Romeo and Juliet) can lead to the generation of numerous different models of the adolescent mind. Whether the novel itself (context of discovery) may be overly simplistic or wrong is irrelevant for scientific progress, as long as the ideas from the novel are translated into testable theories (context of justification), living up to the stricter principles of science (e.g. falsifiability).

The term *heuristic* is of Greek origin meaning “serving to find out or discover.” Traditionally heuristics are considered to be part of the context of discovery (Polya, 1957). Indeed, on several occasions Albert Einstein used falsified theorems as heuristics to generate a novel hypothesis (e.g. Einstein, 1905). However, it has been recognized that within the social sciences heuristic models provide a ‘context of discovery’ that is less trivial. That is because these heuristic models in social science, such as the dual-systems (Casey, 2014, Luna et al., 2013, Shulman et al., n.d.) and social information processing (Nelson et al., n.d.) models, have significant impact on budget streams and what type of research is performed and published. Therefore, heuristic models stand with one foot in the ‘context of justification’ (Nickles, 2006) and do require sufficient motivation and argumentation. This becomes even more important if these models are used to communicate the state of science to people outside the field of inquiry (e.g. policy and law-makers). Here, we suggest two criteria that can be used to evaluate the usefulness of heuristic models: *simplicity* and *specificity.* In the next section we will briefly address these criteria and apply them to the models presented in this volume.

“All models are wrong but some are useful”

(Box, 1976)

### 1.2 Simplicity

A heuristic model is by definition reductionistic, balancing simplicity and specificity against usefulness. Where *simplicity* refers to the number of elements or constructs that make up a model, *specificity* concerns the definition of these constructs. A model that is too complex or too unspecific would not be useful because it does not help constrain the hypothesis space, and does not provide clear starting points for research. However, a model that is too simple runs into the danger of being too restrictive in its agenda setting; not leaving enough room for plausible alternative explanations. Finally, a heuristic model that is too specific turns into the hypothesis it was supposed to inspire.

First, lets consider simplicity. In this context Shulman et al. (n.d.) discuss the triadic model proposed by Ernst et al., 2006. This model suggests that besides reward seeking and cognitive control, developmental changes in avoidance behavior, and corresponding neural systems (e.g. amygdala and insula), are necessary to explain adolescent risky decision-making in its complexity. However, Shulman et al. (n.d.) state that there is “*not much evidence to date indicating that the emotion/avoidance system and its developmental trajectory help to explain heightened levels of risk taking in adolescence*”. Thus, according to Shulman et al. it therefore does not warrant further review, nor is it considered a relevant extension of the DS model. In other words they prefer a simpler 2 systems model to a more complex 3 systems model.

There is reason to believe that in this case absence of evidence may be taken for evidence of absence. First, for most canonical behavioral tasks it is difficult to distinguish the contribution of increased approach from that of reduced avoidance on choice behavior (Luciana and Segalowitz, 2014). Second, considering the development of risk preferences we see that adolescents are consistently risk-averse, not risk-seeking, just less so than adults (for review see Defoe et al., 2015). Fourth, when we turn to the adult neuroscience literature there is ample evidence for involvement of the proposed avoidance network in risky decision-making (e.g. Bossaerts, 2010, Preuschoff et al., 2008, Xue et al., 2010). In sum, we consider a role for avoidance-related processes in risk taking very plausible. For these reasons such a system should not be easily dismissed, but considered a valuable part of a heuristic model.

If we turn to the SIP model we can see that this has so many moving parts (and their connections) that, even though they are organized in three nodes, it is challenging to derive specific hypotheses about neural mechanisms underlying social behavior. Still, there is some value in embracing this complexity and admitting to our lack of knowledge about which of the many component processes is most relevant for understanding developmental changes. In contrast to this view, the more restricted DS model may interpret changes in behavior related to social context directly in terms of changes in reward related striatal activity (e.g. Chein et al., 2011). However, such a strategy may lead to overlooking that the changes in striatal activity are not due to changes in modulations of the striatum by regions involved in social cognition. For example, functional connectivity analyses have shown that the temporal parietal junction (TPJ) may modulate value computation in the striatum (Carter et al., 2012, Hare et al., 2010, van den Bos et al., 2013), and this social brain region is also known to show significant development across adolescence (Burnett et al., 2011, Güroğlu et al., 2011).

These examples illustrate that one should be aware of being too simplistic given its negative consequences for knowledge production (for similar arguments see Greenwald et al., 1986, Greenwald, 2012). This is particularly dangerous in context of neuroimaging studies when one may decide to focus at only a handful highly selected ROIs, justified in part by referring to a heuristic model. As a result, research is actively ignoring parts of the data that are collected, which will not even allow for serendipitous findings. For instance, based on the DS model this may lead to underreporting on the roles of the insula or TPJ in adolescent risk-taking. On the other hand, pursuing a heuristic that is based on only very little evidence will in the worst-case lead to several non-confirmatory studies. Thus, we would argue that in most occasions it is probably better to err on the side of openness, particularly when there are alternative hypotheses that are plausible and relatively well defined.

### 1.3 Specificity

Too little specificity diminishes the usefulness of a heuristic model, simply because it allows for a potentially unlimited number of hypotheses that are consistent with it. Additionally, when moving to the domain of hypothesis testing conceptual specificity is strongly related to classic issues of validity (construct, internal, external; MacKenzie, 2003), which requires both clarity of concepts and clear description of the relationship between the concepts. Here we will argue that for both heuristic models (DS and SIP) there is still room, and need, for further specification of the concepts. Given that the DS model is more specific in its descriptions compared to the SIP model we will use it as an example but most of the following general points also apply to the SIP and other heuristic models. Consider the following statement;

“Specifically, it proposes that risk-taking behaviors peak during adolescence because activation of an early-maturing incentive-processing system (the “socioemotional system”) amplifies adolescents’ affinity for exciting, novel, and risky activities, while a countervailing, but slower to mature, “cognitive control” system is not yet far enough along in its development to consistently restrain potentially hazardous impulses”

(Shulman et al., n.d.)

Some of the notions in this quote are further specified in the paper, such as the regions associated with each system. Still, other important notions, such as reward sensitivity, remain unspecified and thus leave quite some room for interpretation. As we will try to show, one of the consequences of this lack of specificity is that the model allows for the generation of a virtually unlimited number of testable hypotheses, which may be inconsistent with each other but that are all consistent with this general claim.

For example, reward sensitivity does not describe a cognitive or a neurobiological process. Still it is suggested that activity in the socio-emotional system (including ventral striatum) is associated with increased reward sensitivity. However, we striatal activity can represent different processes and reward sensitivity can mean different things. Thus, the consequence of such a definition is that any finding that shows greater ventral striatal activity in teenagers compared to other groups might count as evidence for the DS model, independently of what the underlying mechanism is. Thus, one study may claim that adolescent risk is caused by socio-emotional process X (e.g. outcome processing), whereas another claims it is caused by socio-emotional process Y (e.g. reward anticipation). However, even if these are very different processes the results (increased striatal activity) are still be consistent with the general claim of the DS model.

A related issue is that the networks of brain regions identified by the heuristic models compromise a very large set of structures that are involved in very different functions. This inaccuracy with respect to the anatomy is particularly relevant in case of the striatum, which is a very heterogeneous structure (Haber and Knutson, 2010). For example, finding a certain pattern of activity in the nucleus accumbens versus the dorsal medial striatum has very different functional implications (e.g. van den Bos et al., 2015). However, currently it seems that finding a trend in either one would be consistent with the DS model, irrespectively of specific anatomical localization and associated function.

Furthermore, risk-taking itself is not well defined and is currently measured using various tools (self-report and behavioral measures) that differ on many aspects and whose outcomes measures are known to be uncorrelated (e.g. BART and IGT; for review see Schonberg et al., 2011). This inconsistency raises the question how well each of these measures is related to real-world outcomes (construct validity). Again, the bottom line is that finding a developmental trend in any one of these measures will in principle validate the model.

Interestingly, both papers present the notion that behavior or the activity within the affective/socio-emotional system may “vary as function of stimuli, context or task demand” as important revisions of earlier versions of the models. However, it is still largely unknown what these “context” or the “task demands” are. Richards et al. (2013) provide probably the best overview of all these different effects in context of reward based paradigms, but this paper also illustrates the difficulty of synthesizing these specific effects based on to the more abstract level of heuristics. The problem is that, without further specification, such a general statement about possible context effects introduces more degrees of freedom and questions the generalizability of existing results (external validity).

In sum, the large degrees of freedom due to lack of specificity makes the heuristic models in a practical sense un-falsifiable. Strictly speaking, for a *heuristic model* that is not a problem. Heuristics should guide the formulations of novel hypotheses, and those hypotheses, not the heuristic, should be falsifiable. Still as we pointed out, usefulness can and should be questioned. Indeed the large number of degrees of freedom also leads to limited constraints on the hypothesis space and therefore limits the usefulness of these heuristics. In addition, as MacKenzie (2003) pointed out, that when concepts are not well defined this not only leads to weak theoretical rational for hypotheses but also to misspecification of the measurement model (e.g. which task to use, the localization and interpretation of brain activity etc.)

**Fig. 1:**
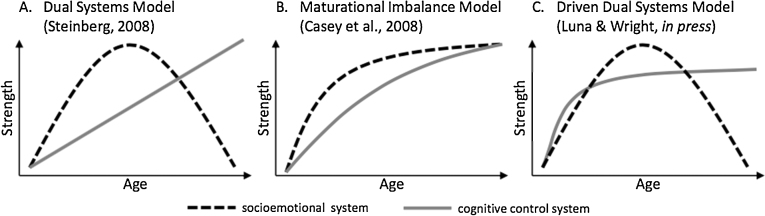
From Shulman et al., n.d.

Given the lack of specificity in constructs, such as system strength or maturation, the three different DS models presented in Fig. 1 are in a practical sense indistinguishable. That is, it is hard to think of a testable hypothesis that could be derived from one model but not the others, or data that would be consistent with one but not another model (indeed Shulman et al., n.d. acknowledge that all models are based on the same data). As a result it is therefore it is at this stage probably also not useful to discuss the relative value of each model compared to the others.

So where do we go from here? As pointed out there is room for improvement in the heuristic models. However, we argue that at this stage there are three recommendations that could have more direct positive impact on developmental neuroscience than trying to improve our heuristic models. First, we should recognize the specific role of heuristic models and stop using heuristic model as cognitive models that can be tested, but rather use them to derive testable hypothesis. We will illustrate how this misuse may lead to conceptual confusion and provides the illusion of progress. Second, in order to achieve real progress we need to think more deeply in terms of cognitive processes and cognitive neuroscience models. That is, a strong focus on cognitive processes will facilitate understanding the relation between changes in brain and behavior, and help understand how different experimental tasks and studies interconnect.

Third, we should recognize that a substantial amount of the work that we do is exploratory not confirmatory. We cannot always be specific, or generate a proper hypothesis, simply due to lack of knowledge. However, exploratory science should not be seen as a lesser form of science, but it should be acknowledged that null hypothesis significant testing (NHST) statistics are not appropriate, and *p* values not meaningful, in this context. Instead we should use exploratory data analyses techniques like factor analyses and cross-validation. Developmental cognitive neuroscience will thrive when we rely on, and integrate, both the empiricist (correlational) and rational (hypothesis testing) modes of science (Cronbach, 1957).

In the next section we address the first and second point more extensively, the third point is beyond the scope of this paper and has been argued for more extensively by others (e.g. Biswal et al., 2010).

## 2 Cognitive models and processes

“Neuroscience is rapidly accumulating a wealth of data at multiple levels ranging from molecules to cells to circuits to systems. However, in the absence of cognitive theory, this effort runs the risk of mere “stamp collecting”, or the tendency to catalog the phenomena of the brain without gaining understanding or explanation.”

(Frank and Badre, 2015)

### 2.1 Consistency Fallacy

The heuristic models have inspired many plausible, yet idiosyncratic hypotheses. That is, most studies in developmental cognitive neuroscience have used a unique set of age comparisons, unique experimental tasks, and unique statistical models for imaging analyses. For instance, consider the studies relevant to adolescent reward sensitivity mentioned in the Shulman paper. This list contains studies on risk and intertemporal choice, studies that focus on the moment of choice or moment of outcome, and studies that have no behavior at all. Many developmental trends are localized in many different brain regions using various methods of analyses (see Richards et al., 2013 for an overview). Even though it is clear that these studies report on very different types of processes associated with activity in different brain regions, their results may appear consistent with each other because they are all consistent with parts of the same heuristic model.

However, this sense of accumulating evidence is misleading and the result of the consistency fallacy (Coltheart, 2013). That is, claiming support for a theory when you report results that are consistent with the theory. However, “*When neuroimaging data from an experiment are consistent with predictions from a particular theory, this cannot be offered as evidence in support of that theory unless it can be shown that there were possible other outcomes of the experiment that are inconsistent with the theory—outcomes that would have falsified predictions from the theory had they been obtained*” (Coltheart, 2013).

As we pointed out above due the level specificity of the constructs and the option to invoke non-described variables (context), it is practically impossible to obtain outcomes that would have falsified the theory.

Note, however, that this issue arises because experimental data are directly related to a heuristic model, which is simply a category mistake. The heuristic model should only be used to generate hypotheses, and these hypotheses should be tested against the data. Of course, that does not mean that new empirical findings can be used to update our heuristic models. It is therefore also very useful to review the numerous studies that were inspired by the heuristic models.

However, at this point we should focus on understanding the relations between the many empirical results of the different studies, rather than evaluating each individually to see whether it provides affirmation of a model on a more abstract level. The danger is that if we are only able to relate our results to each other on such an abstract level we are basically “stamp collecting” (Frank and Badre, 2015). That is, performing many excellent studies but not furthering understanding or explanation because there is no real framework to connect the different results. Here we suggest that progress will be possible by developing more comprehensive cognitive models and the specification of processes (and experimental paradigms).

### 2.2 What makes a good cognitive neuroscience model?

Historically cognitive models have been labeled as symbolic models. That is, they consist of a symbol system, which can thought of as set of distinct mental representations (Newell and Simon, 1976, Putnam, 1960). This symbols are manipulated on the basis of explicit rules, which can be though of as cognitive processes. Busemeyer and Diederich (2010) suggest that in contrast to conceptual (heuristic) models, cognitive models aim to explain one or more of the basic cognitive processes, or explain how these processes interact. That is, cognitive models are derived from the basic principles of cognition. The main advantage of cognitive over heuristic models is that they allow us make logically valid predictions. Moreover, when mathematically formalized, cognitive models can also make quantitative predictions. Of course, this ability to make precise predictions about the results of experimental manipulations comes at the cost of a greater specificity, which may lead to the fragmentation of a research field.

David Marr and Tomaso Poggio (1976) proposed that cognitive neuroscience models should consist of three levels of analyses; a computational, an algorithmic and a physical implementation level. The computational level is based on an abstract problem analysis, which means to decompose the task that is to be performed into its constituent parts (akin to a cognitive model). The algorithmic level specifies a formal process that translates input to a cognitive system to output (a process model). Finally, the implementation level refers to how the cognitive process is implemented in the brain. It is clear that certain problems can be solved by an endless number of different algorithms (different cognitive theories)(Churchland and Sejnowski, 1988), however we are mainly interested which one of those represents the human mind. Imposing the constraint that the model must be implemented in the human neural architecture has been extremely helpful in constraining the search space of algorithmic (process) models (Forstmann et al., 2011).

Taken together, we suggest that a good cognitive neuroscience model should have three building blocks: (1) It needs a good cognitive model of the task that is to be performed but this cognitive model should be broad enough to be generalizable, (2) it has to specify the processes that translates input (stimulus) into output (behavior) and, (3) it has to account for the neurobiological constraints in terms of the functional and structural organization of the brain. This further necessitates that (4)the theory makes predictions about the relation between process and specific neural measures. One example for a successful implementation of such a theory is the case of reinforcement learning. Based on behavioral findings in bees and based on knowledge about dopamine neurons in the bee brain Montague and colleagues (Montague et al., 1995) developed a neurophysiological plausible model of bee foraging. The model was inspired by an RL algorithm that was borrowed from artificial intelligence and that learns to predict future reward value based on reward prediction errors (the difference between the predicted and the experienced reward). The model proved to be extremely successful in providing a mechanistic explanation for foraging behavior in bees and triggered a wealth of model-based studies in primates and humans that explain the neurophysiological mechanisms underlying reinforcement learning (Schultz et al., 1997). What we gain from such a model is the ability to predict behavior and brain mechanisms on a qualitative level. If the model is formulized, which at some level of complexity has to be, we can even use it to make quantitative predictions about our outcome variables, such as neural activity (Busemeyer and Diederich, 2010).

One issue that arises when moving from a heuristic to a cognitive model is that the level of specificity of the cognitive model is much higher. That is, a certain cognitive model may only be applied to a limited range of scenarios or tasks (e.g. description based risk taking). For example, instead of subsuming choice processes during delay discounting and risky decision-making under the umbrella construct of reward sensitivity we now have to specify the actual processes that allow individuals to integrate their subjective perception of time, probability and value when making value-based decisions. The advantage of this approach is that for each of these processes we can independently establish the developmental trajectories and thus get a more detailed understanding of the mechanisms underlying developmental change in behavior. For instance, using this framework two recent papers have identified to possible processes related to developmental differences in reinforcement learning; (1) increased activity in response to positive (but not negative) prediction error in the striatum (Cohen et al., 2010), and (2) differences in the value updating process associated with developmental changes in functional striatum-prefrontal connectivity (van Den Bos et al., 2012). Finally, capitalizing on existing cognitive models may support more meaningful generalizations across multiple tasks and scenarios. In the following we will shortly discuss two such models. One of them originates from cognitive neuroscience (value-based decision-making theory (Rangel et al., 2008)), and one is a genuine developmental theory (the interactive specialization model (Johnson, 2011)).

The value-based decision-making model (see Fig. 1; Rangel et al., 2008) summarizes the five component processes of value-based decision-making: (1) The individual has to compute a *representation* of the decision problem that is, the internal and external states as well as the potential courses of actions. (2) The decision-maker has to assign a (subjective) *value* to the available actions. (3) The different values have to be compared to select an *action.* (4) After the decision the *outcome* has to be evaluated. (5) Finally, the outcome evaluation has to be used to refine predictions (*learning*).

Note that a key part of this framework is that cognitive processes operate on values and expectations that are constructed and intrinsically subjective. Importantly the model also provides a framework to compare and integrate different types of values, such as social and monetary outcomes, on a common scale (for example van den Bos et al., 2013). As such it provides a unique quantitative framework for understanding of how social context modulate specific decision processes in adolescence. Furthermore, this taxonomy of decision processes can be useful in dissociating developmental differences in the different component processes during decision-making. As such it is helpful to identify which of these processes are developing and which are important for understanding adolescent specific choice patterns. Additionally, this can help identify the developmental specific effects of affective states on decision processes (e.g. it may effect learning but not value comparison), another topic which is particularly relevant for understanding adolescent behavior. Finally, it could provide a very useful model for integrating developmental differences across a range of decision paradigms and might thus allow inferences across multiple domains (e.g. risky, social and intertemporal decision-making; (Glimcher and Fehr, 2013).

Here we consider what it means to apply such a modeling perspective to understanding the neurocognitive development of simple reward-based decision making. The current literature on developmental differences in reward processing shows several inconsistencies. Richards et al. (2013) have provided an excellent mapping of all these different results, breaking them down by type of task (passive reward, instrumental reward and decision-making) and phase of task (e.g. decision, anticipation and feedback). The goal of the paper, from the perspective from the heuristic models, is to figure out under which circumstances one finds specific activation patterns (e.g. adolescent peak in striatum) for specific phases of the tasks (e.g. feedback processing). However, from a cognitive modeling perspective one would ask a different type of question; how could greater ventral striatal response during outcome processing, in any of these tasks, be meaningfully related choice behavior? Simply correlating activation levels with some external measure of risk would not inform us about the underlying mechanism. The striatal response happens after the decisions are made so it is unclear how it can *prima facie* be related to the choice process itself. Moreover, what if the peak in outcome related activity is reported in relation to passive reward task or instrumental tasks in which there is a response but no choice? The heuristic models don’t force us to further investigate these mechanistic questions because they care less about *how* things happened and more about *what* happened (increased ventral striatal activity). In contrast, the cognitive modeling perspective forces us be more precise about the underlying mechanisms, connecting stimulus to response.

According to two paradigmatic value based decision models, (e.g. reinforcement learning (Niv and Schoenbaum, 2008) or predictive coding (Friston, 2010), outcomes are always evaluated in relation to prior expectations. This suggest that adolescents may show different outcome related responses because have (1) different subjective expectations (e.g. differences in probability weighting function (Gonzalez and Wu, 1999), or (2) different value functions (Kahneman and Tversky, 1979)). For instance adolescents could subjectively think the risky, but positive, outcome was less likely to happen (even if objective probability was the same). In that case the increased activity could reflect greater positive surprise (positive prediction error). Although this explanation is able to make inferences about the decision phase based upon how outcomes are processed, this would not directly explain increased risk taking. That is, a smaller subjective probability should in principle be associated with less risk taking.

Another alternative is that adolescents assign a higher subjective value to the actual outcome that they receive, regardless of their expectation (for similar argument see (Vaidya et al., 2013). Differences in the shape of the value function (which translates objective to subjective value,) could mean that for adolescence the large reward associated with risky option indeed has a higher subjective value. For instance, it is possible that money has more (subjective) value for adolescents because they have a smaller budget than adults, but more spending freedom/opportunity than children. Note that if this were true, we would expect adolescents to take more risks when presented with the same set of gambles, even if they would have similar risk preferences as adults.

**Fig. 2:**
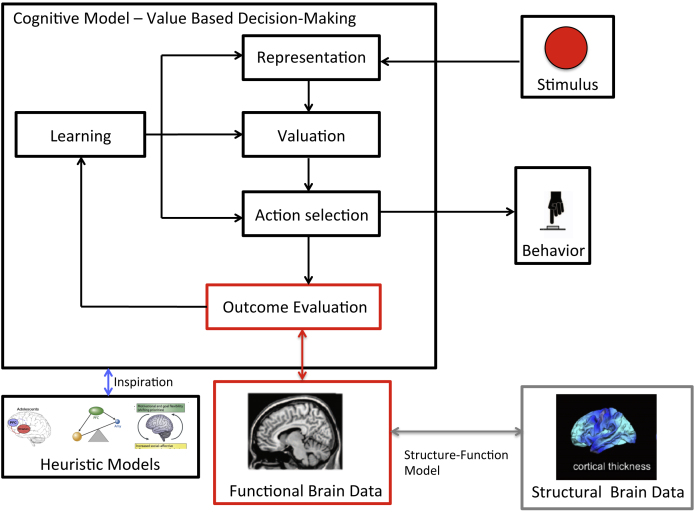
Schematic overview of relation between heuristic models, process models and data. The example highlights how functional imaging data related to outcome evaluation may relate to other processes involved in value-based decision-making.

Unfortunately, based on the existing data it is hard to tell to what extend subjective value or subjective probability differences play a role in developmental changes in brain activity and behavior. However, the field of decision neuroscience provides plenty of tasks and analytical tools to measure these constructs (for overview see Glimcher and Fehr, 2013), which can be easily applied to understand developmental processes. Again, these frameworks and accompanying tools can now also be used to estimate difference in social values and differential impact of affect across development. Thus, although we are not yet able to synthesize existing results, and thus only able to raise new questions, we hoped to illustrate that these questions, and related constructs, may help to better understand the underlying processes. We are confident that when we have a more detailed understanding of the development of component processes in value based decision-making we can (1) better predict when an where we will find developmental differences, and (2) how processes of different measured in different tasks are related. This is probably a quicker route that trying out numerous task variations and see if a pattern will emerge in the data. Note that this approach does not necessarily requires advanced mathematical modeling, but rather a more detailed account of task relevant processes and how they are related (see for instance Fig. 2). Furthermore, when we have a better idea of which process we evoke with our task we have to rely less on reverse inference when interpreting our imaging data.

Finally, we would like to stress that the interactive specialization model (Johnson, 2011) might provide an important link between changes on the social behavioral level and changes on the neurobiological level. For example, one could imagine that developmental periods that are characterized by specific interaction types (infant–caregiver interactions, peer interactions in adolescents) might trigger activity-dependent interactions between regions that lead to a sharpening of their respective functions and response properties. On the other hand, social behavioral changes should be accompanied by changes in the functional connectivity between several networks (Johnson, 2011). This model seems to fit in very naturally with the account provided by Nelson and colleagues (n.d.). We realize that integrating this might be a complicated endeavor. However, being able to specify the processes that link neurobiology to changes in social behavior in different age groups referring to interactive specialization might be useful.

## 3 Conclusion

The papers by Shulman et al. (n.d.) and Nelson et al. (n.d.) provide interesting and useful heuristic frameworks to think about the development of motivational and cognitive control processes as well as the development of social behavior. As outlined above heuristic models have a great value in setting research agendas and stimulating the evolution of cognitive neuroscience theories of development. However, heuristic theories have limited predictive power: they do not allow making precise predictions about how development affects the underlying cognitive and neurobiological processes. It is the natural evolution for a new field to shift from the heuristic phase of agenda setting to a more critical hypothesis testing and falsification phase (Eysenck, 1997). Here we argued that in order to advance the field of developmental cognitive neuroscience it is time to start making this transition. We should aim at improving our heuristic models but at the same time we should shift focus toward developing more precise, but testable, cognitive neuroscience models.
